# Satisfaction and perceptions about aspects of the city that affect health, by socioeconomic status, 2010-2019, in Lima

**DOI:** 10.17843/rpmesp.2022.391.9888

**Published:** 2022-03-31

**Authors:** Victoria Cavero, Akram Hernández-Vásquez, J. Jaime Miranda, Patricia Alata, Mariana Alegre, Francisco Diez-Canseco

**Affiliations:** 1 CRONICAS Centro de Excelencia en Enfermedades Crónicas, Universidad Peruana Cayetano Heredia, Lima, Peru. Universidad Peruana Cayetano Heredia CRONICAS Centro de Excelencia en Enfermedades Crónicas Universidad Peruana Cayetano Heredia Lima Peru; 2 Lima Cómo Vamos (LCV), Lima, Peru. Lima Cómo Vamos (LCV) Lima Peru

**Keywords:** Urban Health, Environmental Health, Public Health, Quality of Life, Personal Satisfaction, Healthcare Disparities, Public Policy, City Planning, Latin America, Surveys and Questionnaires

## Abstract

We aimed to characterize the satisfaction and perceptions of the residents of Lima about different aspects of urban life that can affect their quality of life and health, identifying differences by socioeconomic status (SES) and changes over time. A secondary data analysis of the “Lima Cómo Vamos” survey was conducted between 2010 and 2019. Results are reported through percentages, with differences between SES for each year and between years. In 2019, satisfaction and perceptions were mostly unfavorable, and have decreased by up to 30% over time. People with lower SES had more unfavorable evaluations and with greater reductions over time. This dissatisfaction and unfavorable perceptions reveal deficiencies in public services and urban conditions that could negatively affect the quality of life and health, making it necessary to design and implement policies that reduce socioeconomic gaps and improve the health of Lima citizens.

## INTRODUCTION

The world’s urban population has quadrupled since 1950, making Latin America one of the most urbanized regions ^1^. This rapid growth, often devoid of social policies and planning, has resulted in cities with major disparities in access to public services, housing, transportation, security and recreation, negatively impacting the poorest and most vulnerable populations ^2^ in regards of their quality of life ^3^ and health. Asthma, cancer, obesity, as well as Cardiovascular and immunological diseases, are more prevalent in urban areas ^4^; whereas pollution and limited access to water and waste management services represent health risks ^5^. These consequences, however, are not homogeneous in Latin American cities, where the socioeconomic status (SES) determines the population’s susceptibility to environmental pollution, and where the poorest populations are at greater risk of mortality from respiratory diseases ^6^. Likewise, although access to green space is associated with greater physical activity, mental wellbeing and less pollution, these benefits are limited by conditions that, such as insecurity, undermine the effective use of these areas ^7^.

Metropolitan Lima has also grown with deficient planning and major social inequalities, which is reflected in the gaps in access to health services and drinking water ^8^. Given the potential impact of these conditions on the quality of life and health of the population, it is important to consider them as a key issue in order to strengthen urban public policies^ (^ ^9^. Other surveys in Lima have explored the population’s satisfaction with public services ^10^ or perceptions of corruption ^11^, but not the impact of urban conditions on citizens’ health.

The aim of this study is to characterize the satisfaction and perceptions of Metropolitan Lima residents about different aspects and services of the city that may affect their quality of life and health, identifying differences by SES as well as changes over time, using information from the “Lima Cómo Vamos” (LCV) survey. The analysis of this survey, which has previously supported policies for Metropolitan Lima ^12^, will provide valuable evidence to improve urban public policies that promote wellbeing and prevent negative health outcomes.

**KEY MESSAGES**

Motivations for the study: Living conditions and urban socioeconomic inequalities affect people’s health; therefore, knowing the perceptions and satisfaction of individuals will allow us to prioritize policies that improve their quality of life and health.

Main findings: A review of data from an annual survey of Lima residents revealed very low satisfaction with the city and its public services, as well as unfavorable perceptions of safety and public transportation. These perceptions have worsened over the years, especially in the lower socioeconomic levels.

Implications: These results will allow us to prioritize policies that promote well-being and prevent negative health outcomes.

## THE STUDY

A secondary analysis of the LCV survey was conducted from 2010 to 2019. Since 2010, the LCV survey uses a representative sample (different every year) of 2000 residents from Metropolitan Lima in order to collect data regarding their satisfaction and perceptions with different dimensions of the city (e.g., pollution, transportation, sanitation). The methodology of the survey, applied by the Institute of Public Opinion of the Pontificia Universidad Católica del Perú (IOP-PUCP), is detailed in the supplementary material.

SES was used as an exposure variable to analyze differences in citizen satisfaction and perceptions. The SES was classified into three levels (A/B [higher SES], C and D/E [lower SES]), according to the IOP-PUCP SES calculation methodology.

We selected 14 questions that addressed satisfaction and perceptions with health-related dimensions and that had a sufficient number of respondents in the disaggregation of responses. The questions are from the following dimensions: satisfaction with the city, citizen safety, environment, public spaces, housing and public services. Each of the selected variables correspond to a question in the survey and are presented in Table 1. Those with a Likert scale with five categories were classified into three categories to simplify result interpretation. Although some survey questions have been applied since 2010, others have been added in later years, on the recommendation of a panel of specialists ^13^; therefore, for some variables, the year of comparison is not 2010 but 2013, 2015 or 2016.

**Table 1:** Variables included in the study, including database code, question, and survey comparison years.

Code	Variable of interest	Question	Comparison years ^c^
EG3_X	Satisfaction with the city of Lima as a place to live (yes/no) ^a^	Overall, how satisfied do you feel with Lima as a city to live in?	2010 and 2019
MA1F_X	Satisfaction with Lima’s air quality (yes/no) ^a^	How would you rate your overall level of satisfaction with the following aspects that influence the quality of life of people in Lima?	2010 and 2019
MA1D_X	Satisfaction with noise levels in Lima (yes/no) ^a^	How would you rate your overall level of satisfaction with the following aspects that influence the quality of life of people in Lima?	2010 and 2019
MA1E_X	Satisfaction with Lima’s green spaces (yes/no) ^a^	How would you rate your overall level of satisfaction with the following aspects that influence the quality of life of people in Lima?	2010 and 2019
EP5_X	Satisfaction with Lima’s public space (yes/no) ^a^	Defining public space as places open to all people in the city, how satisfied are you with the public space available in Lima?	2015 and 2019
EP6_X	Satisfaction with the public space where you live (yes/no) ^a^	How satisfied are you with the public space available where you live?	2015 and 2019
MA1C_X	Satisfaction with garbage collection in Lima (yes/no) ^a^	How would you rate your overall level of satisfaction with the following aspects that influence the quality of life of people in Lima?	2010 and 2019
MA1I_X	Satisfaction with Lima’s access and quality of water (yes/no) ^a^	How would you rate your overall level of satisfaction with the following aspects that influence the quality of life of people in Lima?	2016 and 2019
EG6E_X	Satisfaction with health services in Lima (yes/no) ^a^	How would you rate your level of satisfaction with the health services available in the city of Lima?	2010 and 2019
EG7_1	Perception of citizen insecurity as one of the three most important problems of the city (yes/no) ^a^	From the following list, what do you think are the three most important problems affecting the quality of life in the city of Lima?	2010 and 2019
EG7_2			
EG7_3			
EG4_X	Perception that Lima is a safe city (yes/no) ^b^	Currently, regarding violence and crime, would you say that Lima is safe?	2010 and 2019
VS1_X	Perception of safety in the area of residence (yes/no) ^b^	Currently, regarding to violence and crime, would you say that the place where you live is safe?	2010 and 2019
EG7_1	Perception of public transportation as one of the three most important problems of the city (yes/no) ^a^	From the following list, what do you think are the three most important problems affecting the quality of life in the city of Lima?	2010 and 2019
EG7_2			
EG7_3			
MA2_1_1	Perception of vehicular pollution as the city’s most important environmental problem (yes/no) ^a^	Of the issues related to environmental management, what do you think are the three most serious environmental problems?	2013 and 2019

a The questions are asked on a scale of 1 to 5, with 1 being “Very dissatisfied” and 5 being “Very satisfied”. It was categorized as “Yes” when the person expressed being satisfied (4-5) and “No” when the person responded being dissatisfied (1-2) or indifferent (3).

b The questions are asked on a scale of 1 to 5, with 1 being “Not at all sure” and 5 being “Very sure”. It was categorized as “Yes” when the person reported feeling confident (4-5) and “No” when the person responded feeling insecure (1-2) or neither confident nor insecure (3).

c A comparison was made between the oldest and most recent year according to the availability of each question.

The sociodemographic characteristics of the participants were described using frequencies. Satisfaction and perceptions were reported as percentages with 95% confidence intervals (95% CI) for the two comparison years. In addition, differences between SES for each year were determined using the chi-square test. We analyzed differences in percentage points (PP) and their 95% CI for satisfaction and perceptions according to SES between the oldest and most recent year for each question using a linear combination of the compared estimates. According to the survey data sheet ^13^, the estimates do not require weighting factors since the sample distribution was proportional to the population distribution according to 2007 Census data. A p-value <0.05 was considered statistically significant. Statistical processing and analysis were carried out in Stata® v14.2.

The databases, with anonymous information from the participants, and the survey data sheet are in the public domain and freely accessible at: https://www.limacomovamos.org/data/. This study was not subject to review by an ethics committee because of the use of open-access secondary data.

## FINDINGS

Characteristics of the respondents for each selected year are described in Table 2. Women represented about 50% of the population during the 10 studied years; each age group represented approximately one third of the respondents. Regarding SES, the proportion of participants in each level has been changing over time, with more respondents in A/B and C than in D/E during the last few years. The estimates along with their 95% CIs are included in the supplementary material.

**Table 2:** Characteristics of the people surveyed in “Lima Cómo Vamos”.

Characteristic	2010 n (%)	2013 n (%)	2015 n (%)	2016 n (%)	2019 n (%)
Sex					
Male	956 (49.8)	920 (47.9)	923 (48.1)	925 (48.2)	921 (47.9)
Female	964 (50.2)	1000 (52.1)	997 (51.9)	995 (51.8)	999 (52.1)
Age group (years)					
18-29	634 (33.0)	647 (33.7)	650 (33.9)	649 (33.8)	555 (28.9)
30-44	632 (32.9)	619 (32.2)	621 (32.3)	623 (32.5)	611 (31.8)
45 or more	654 (34.1)	654 (34.1)	649 (33.8)	648 (33.7)	754 (39.3)
Socioeconomic status					
A/B	486 (25.3)	72 (3.8)	743 (38.7)	707 (36.8)	700 (36.5)
C	857 (44.6)	1023 (53.2)	676 (35.2)	704 (36.7)	747 (39.0)
D/E	577 (30.1)	825 (43.0)	501 (26.1)	509 (26.5)	469 (24.5)
Interdistrict area of residence					
Central Lima	728 (37.9)	504 (26.2)	504 (26.2)	504 (26.3)	472 (25.9)
Eastern Lima	424 (22.1)	512 (26.7)	512 (26.7)	512 (26.7)	528 (29.0)
Northern Lima	384 (20.0)	504 (26.3)	504 (26.3)	504 (26.2)	536 (29.3)
Southern Lima	384 (20.0)	400 (20.8)	400 (20.8)	400 (20.8)	288 (15.8)

A/B: corresponds to the highest socioeconomic levels.

D/E: corresponds to the lowest socioeconomic levels.

Central Lima: Lima Cercado, Breña, Rímac, La Victoria, Lince, Jesús María, Pueblo Libre, San Miguel, Magdalena del Mar, San Isidro, Miraflores, Barranco, Santiago de Surco, Surquillo, San Borja and San Luis.

Eastern Lima: San Juan de Lurigancho, Ate, Chaclacayo, La Molina, Lurigancho-Chosica, El Agustino, Santa Anita and Cieneguilla.

Northern Lima: Ancon, Carabayllo, Comas, Independencia, Los Olivos, Puente Piedra, San Martin de Porres and Santa Rosa

Southern Lima: Chorrillos, San Juan de Miraflores, Villa María del Triunfo, Villa El Salvador, Lurín, Pachacámac, Punta Hermosa, Punta Negra, San Bartolo, Santa María del Mar and Pucusana

### Satisfaction with the city, the environment and the public services provided

**Figure 1:**
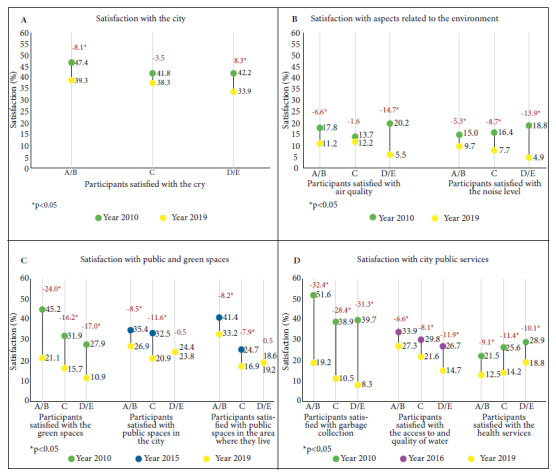
Satisfaction with the city, the environment and public services provided, by socioeconomic level and year, Lima, Peru

Satisfaction with the city decreased by 5.8 PP between 2010 and 2019 (43.3% to 37.5%; p<0.001). Regarding SES, satisfaction in the A/B and D/E levels decreased by approximately 8 PP. In 2010 and 2019, satisfaction by SES was similar (Figure 1).

*Satisfaction with aspects related to the environment*

The proportion of people satisfied with the air quality decreased by 6.5 PP between 2010 and 2019 (16.7 to 10.2%; p<0.001). Regarding SES, there were changes in levels A/B and D/E, with a greater reduction in level D/E. There were differences in the satisfaction between SES levels in 2010 (p=0.005) and 2019 (p=0.001). In 2010, D/E was the stratum most satisfied with air quality, but in 2019 it was the least satisfied.

The proportion of people satisfied with noise levels decreased by 9.1 PP between 2010 and 2019 (16.8 to 7.7%; p<0.001). This satisfaction decreased in all SES levels, although more so in D/E. While in 2010 there were no differences between SES levels (p=0.238), in 2019 satisfaction was twice as high in A/B as in D/E (9.7 vs. 4.9%; p=0.01) (Figure 1).

*Satisfaction with green spaces and public spaces*

The proportion of people satisfied with green spaces halved between 2010 and 2019, from 34.1 to 16.5% (p<0.001) and decreased in all SES levels. In both years there were differences between SES levels, finding that A/B were more satisfied, especially in 2019, where A/B doubled the satisfaction of D/E (21.1 vs. 10.9%).

Satisfaction with public spaces in Lima and public spaces in the area of residence decreased by approximately 7 PP between 2015 and 2019 (31.5 to 23.8%; p<0.001; and 29.6 to 23.5%; p<0.001). Regarding the SES, we only observed decreases between 2015 and 2019 in levels A/B and C. In both years there were differences between SES levels, with A/B being the most satisfied. Likewise, in level A/B more people were satisfied with public spaces in their area than in Lima, both in 2010 (41.2 vs. 35.4%) and in 2019 (33.2 vs. 26.9%). The opposite was true for the C and D/E levels, more people were satisfied with public spaces in Lima than in their area in 2010 (C: 24.7 vs. 32.5%; D/E: 18.6 vs. 24.4%) and 2019 (C: 16.9 vs. 20.9%; D/E: 19.2 vs. 23.8%) (Figure 1).

*Satisfaction with public services*

Satisfaction regarding garbage collection decreased to one third between 2010 and 2019, going from 42.3 to 13.1% (p<0.001), and being similar in all SES levels. In 2010 and 2019 there were differences between SES levels, with A/B being the most satisfied; mainly in 2019, when it doubled the satisfaction of D/E (19.2 vs. 8.3%).

Satisfaction with water access and quality decreased by 8.4 PP between 2016 and 2019 (30.5 to 22.0%; p<0.001) and dropped in all SES levels, although more in D/E compared to A/B (-11.9 vs. -6.6%). In addition, A/B was always the most satisfied stratum; especially in 2019, when it doubled D/E (27.3 vs. 14.7%).

Satisfaction with health services dropped by almost half between 2010 and 2019 (25.6% to 14.7%; p<0.001), decreasing in all SES levels. Those in the D/E level were consistently more satisfied than in other SES levels (Figure 1).

### Perception of the city’s main problems

The two problems perceived as most important for the quality of life in Lima in 2010 and 2019 were citizen insecurity and public transportation.

*Citizen insecurity*

The perception of citizen insecurity as one of Lima’s main problems increased by 8.7 PP between 2010 and 2019 (73.5 to 82.2%; p<0.001); although this increase was only in the C and D/E levels (C: p<0.001; D/E: p=0.001). By SES level, the proportion of people who considered it a main problem was always higher in A/B (81.5% and 85.1%) than in other SES levels.

The perception of Lima as a safe city decreased by 6.4 PP between 2010 and 2019 (17.7% to 11.4%; p<0.001), dropping in all SES levels, but more so in D/E. In 2010, more people in D/E shared this perception; but in 2019 no differences were found between SES levels.

**Figure 2:**
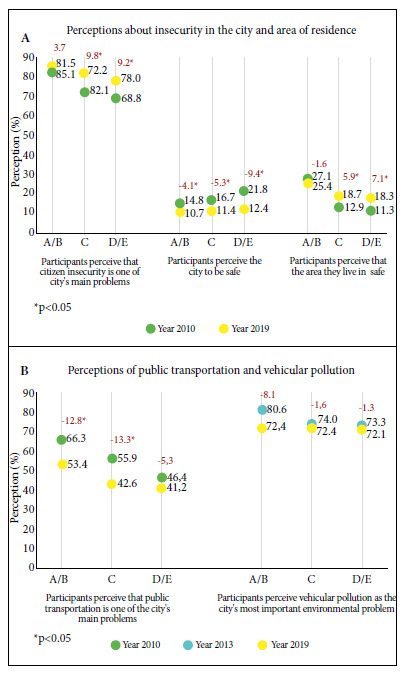
Perception of the main problems of the city and the environment, by socioeconomic status and year, Lima, Peru.

The perception of the area of residence as being safe increased 5.1 PP between 2010 and 2019 (16.0 to 21.1%; p<0.001). By SES level, the feeling of safety increased only in C and D/E. In addition, people in the A/B level were always the ones that felt the safest in their place of residence (Figure 2).

*Public transportation*

The perception of public transportation as a major problem in Lima decreased by 9.5 PP between 2010 and 2019 (55.7% to 46.2%; p<0.001), decreasing in SES levels A/B and C. In both years more people in A/B than other levels considered it an important problem. No differences were observed in the perception of vehicle pollution as the most serious environmental problem (Figure 2).

## DISCUSSION

The study sought to characterize the satisfaction and perceptions, including changes over time, of Metropolitan Lima residents on aspects of urban life that could affect their quality of life and health, with emphasis on differences by SES levels. The main results are three. First, in 2019, the vast majority of respondents were dissatisfied and perceived unfavorably different services and features of the city. Second, although many of these ratings were already low in the first year (usually 2010), most decreased even more. Third, ratings were usually not homogeneous by SES level, with people in the D/E level having worse ratings and greater drops in satisfaction over time. These findings suggest that improvements in Lima’s human development indicators ^14^ did not increase the satisfaction of its citizens and that some gaps between SES levels, instead of narrowing, may have increased.

Compared to cities such as Santiago de Chile ^15^ or Bogotá ^16^, the satisfaction of Lima residents with the city and its services is much lower. Satisfaction with Santiago de Chile in 2007 was over 80%, and has increased over the years ^15^; while in Lima it does not reach 40%. In Bogotá, satisfaction with water and garbage collection services was 84% and 65%, respectively ^20^; considerably higher than the 25% and 15% in Lima. The recognition of the right to a better urban environment has been in place for many years in other cities in the region before Lima, which may have resulted in better conditions and services, leading to more satisfied citizens.

Of all the issues explored, the highest level of dissatisfaction was related to noise and air quality, which had the worse ratings over time and among the poorest. This is consistent with the fact that noise pollution in Lima exceeds national environmental quality standards for noise ^17^. However, available data on air quality shows that it has improved by 60% in the last decade, although the values of particulate matter (PM10 and PM2.5) still exceed the maximum permitted levels ^18^. Another relevant result is the low satisfaction with green spaces, which reflects its great deficit, since Lima is among the 5% of capital cities with the fewest green spaces in the world ^19^.

Satisfaction with water and garbage collection services is low as well. First, Lima suffers from chronic water stress, with 20% of Lima residents lacking daily access to drinking water ^20^. The higher dissatisfaction in the D/E SES levels confirms the urgency of policies to reduce this gap in access to water. Second, Lima is the city that produces most of the garbage in Peru, but has several municipalities that do not have waste management programs or adequate public cleaning services ^21^. Satisfaction with health services is also low; it was 15% in 2019 and has decreased across all SES levels; being almost a quarter than what was reported for Bogota in 2019 ^16^. The low quality of health services has been reported by the Ombudsman’s Office ^22^, and is associated with supply and care issues. Finally, although crime in Lima has decreased over the years ^23^, insecurity continues to be a priority problem. Likewise, greater security in the area of residence could indicate greater trust with neighbors or better management by district municipalities.

It is essential to implement policies to reduce the gaps and improve the services, as well as policies to address the studied issues due to their potential impact on health. Examples of these actions would be to reduce air pollution, which is associated with higher cardiorespiratory mortality in Lima ^24^, and to improve drinking water services to mitigate the risk of gastrointestinal diseases, dengue or accidents when carrying water to the home, especially in the lower SES levels ^25^.

The strength of this study is that we analyzed a survey applied annually, for a decade, with the same instruments and methodology, to representative samples of a megacity. One limitation is that not all the 2019 results could be compared with those of 2010, since some questions were included in later years. However, comparisons were almost always made with the year 2010, and for the other cases there was enough difference in years to expect changes over time. On the other hand, the Likert scales were recategorized from five to three categories, in order to simplify the interpretation of the results. Although there is risk of incurring in non-differential measurement error, the annual reports of the LCV survey also use this recategorization.

Our results show high and increasing dissatisfaction with different aspects of the city, as well as gaps between SES levels that widen over time. These results can guide the priorities of policymakers, who tend to focus more on improvements regarding material aspects, such as sanitation or public spaces, but, as we have shown, these do not lead to more satisfied citizens. From a public health perspective, it is necessary that authorities guarantee policies that improve urban living conditions, promoting the well-being of citizens and preventing negative health consequences.

## References

[1] United NationsDepartment of Economic and Social Affairs, Population Division (2019). World Urbanization Prospects: The 2018 Revision.

[2] United Nations (2019). The Sustainable Development Goals Report 2019.

[3] Lora E, Powell A, van Praag BM, Sanguinetti P (2010). The quality of life in Latin American cities: Markets and perception.

[4] Flies EJ, Mavoa S, Zosky GR, Mantzioris E, Williams C, Eri R (2019). Urban-associated diseases Candidate diseases, environmental risk factors, and a path forward. Environment International.

[5] World Health Organization (2019). Healthy environments for healthier populations: Why do they matter, and what can we do?.

[6] Romieu I, Gouveia N, Cifuentes LA, de Leon AP, Junger W, Vera J (2012). Multicity study of air pollution and mortality in Latin America (the ESCALA study). Res Rep Health Eff Inst.

[7] Adhikari B, Mishra SR, Dirks KN (2020). Green space, health, and wellbeing considerations for South Asia. The Lancet Planetary Health.

[8] Centro Nacional de Planeamiento Estratégico (2018). Información de brechas de servicios a nivel departamental, provincial y distrital.

[9] UN-HABITAT, World Health Organization (2020). Integrating health in urban and territorial planning:a sourcebook..

[10] Ipsos Public Affairs (2017). Encuesta Nacional de Satisfacción Ciudadana 2017.

[11] Instituto Nacional de Estadística e Informática (2019). XI Encuesta nacional anual sobre percepciones de corrupción. Informe especial preparado para Proética..

[12] Municipalidad de Lima (2016). Plan de desarrollo local concertado de Lima Metropolitana 2016-2021..

[13] Lima Cómo Vamos (2015). Encuesta Lima Cómo Vamos. VI Informe de percepción sobre calidad de vida..

[14] Yufra S, Huerta PE (2019). Reto de la Igualdad: Una lectura de las dinámicas territoriales en el Perú.

[15] Comisión de Estudios Habitacionales y Urbanos (2018). Cuarta Encuesta de Calidad de Vida Urbana..

[16] Bogotá Cómo Vamos (2019). Encuesta de Percepción Ciudadana 2019.

[17] Organismo de Evaluación y Fiscalización Ambiental OEFA (2016). La contaminación sonora en Lima y Callao.

[18] (2019). Lima Metropolitana y el Callao cuentan con un diagnóstico actualizado sobre la calidad del aire.

[19] Watts N, Amann M, Arnell N, Ayeb-Karlsson S, Beagley J, Belesova K (2021). The 2020 report of The Lancet Countdown on health and climate change responding to converging crises. The Lancet.

[20] Instituto Nacional de Estadística e Informática (2020). Perú: Formas de Acceso al Agua y Saneamiento Básico.

[21] Defensoría del Pueblo (2019). Informe Defensorial N.º 181 ¿Dónde va nuestra basura? Recomendaciones para mejorar la gestión de los residuos sólidos municipales..

[22] Defensoría del Pueblo (2017). Informe de Adjuntía N.° 34-2017-DP/AAE. Análisis de los resultados de la supervisión nacional a los establecimientos de salud estratégicos 2017.

[23] Instituto Nacional de Estadística e Informática (2020). Principales indicadores de seguridad ciudadana a nivel regional. Semestre móvil Noviembre 2019-Abril 2020..

[24] Tapia V, Steenland K, Vu B, Liu Y, Vásquez V, Gonzales GF (2020). PM2.5 exposure on daily cardio-respiratory mortality in Lima, Peru, from 2010 to 2016. Environmental Health.

[25] Adams EA, Stoler J, Adams Y (2020). Water insecurity and urban poverty in the Global South Implications for health and human biology. American Journal of Human Biology.

