# Supplementary material for: Satisfaction and perceptions about aspects of the city that affect health, by socioeconomic status, 2010-2019, in Lima
Source: Rev Peru Med Exp Salud Publica. 2022 Mar 31;39(1):83–90. doi: 10.17843/rpmesp.2022.391.9888 (PMC11397593; doi:10.17843/rpmesp.2022.391.9888)
Supplement: Supplementary material. — Available in the electronic version of the RPMESP. [file rpmesp-39-01-9888-s001.pdf]

# MATERIAL SUPLEMENTARIO

**Anexo 1.** Descripción de la encuesta «Lima Cómo Vamos»

| | |
|---|---|
| Dimensiones de la encuesta | La encuesta recoge información sobre la satisfacción y percepciones de los ciudadanos de Lima Metropolitana con las siguientes dimensiones: satisfacción con la ciudad, seguridad ciudadana, movilidad y transporte, medio ambiente, espacios públicos, vivienda y servicios públicos, manifestaciones culturales, deportes y recreación, educación, salud, participación ciudadana, gestión pública y economía, pobreza y desigualdad. |
| Diseño muestral | El diseño muestral es probabilístico, multietápico y estratificado por conglomerados según zonas de la ciudad (Lima Norte, Lima Este, Lima Centro y Lima Sur), con cuotas por sexo y grupos de edad y cuyo marco muestral son las cartografías del Instituto Nacional de Estadística e Informática. El margen de error es de ±2,24% con un nivel de confianza del 95%, asumiendo una heterogeneidad de 50%-50%. El margen de error para cada estrato geográfico es entre ±4,2 y ±5,0%. El nivel socioeconómico se determina de acuerdo al nivel de instrucción del jefe de hogar, el material predominante en los pisos de la vivienda, tenencia de bienes en el hogar (teléfono fijo, computadora, refrigeradora, horno microondas y lavadora) y hacinamiento, según la metodología de cálculo del Instituto de Opinión Pública de la Pontificia Universidad Católica del Perú. |
| Metodología del trabajo de campo | Para la aplicación del estudio, se toman 1920 casos en 240 unidades primarias de muestreo. Estas se dividen en los cuatro estratos correspondientes a las zonas de la ciudad: Lima Norte (Breña, La Victoria, Lima, Rímac, San Luis, Barranco, Jesús María, Lince, Magdalena del Mar, Miraflores, Pueblo Libre, San Borja, San Isidro, San Miguel, Santiago de Surco y Surquillo); Lima Este (La Molina, Ate, El Agustino, San Juan de Lurigancho, Santa Anita, Chaclacayo, Cieneguilla y Lurigancho); Lima Norte (Carabayllo, Comas, Independencia, Los Olivos, Puente Piedra, San Martín de Porres, Ancón y Santa Rosa) y Lima Sur (Chorrillos, San Juan de Miraflores, Villa El Salvador, Villa María del Triunfo, Lurín, Pachacamac y Balnearios del Sur (Pucusana, Punta Hermosa, Punta Negra, San Bartolo y Santa María del Mar). Se aplica un piloteo de 30 encuestas y tras las correcciones se realiza el trabajo de campo de forma presencial en hogares por un periodo aproximado de un mes. |
| Estrategias que aseguran la calidad de los datos recolectados | El 30% de las encuestas aplicadas son supervisadas a través de reentrevista. |

**Anexo 2.** Satisfacción y percepciones sobre aspectos de la calidad de vida urbana de los residentes en Lima Metropolitana, según niveles socioeconómicos y años, en la encuesta «Lima Cómo Vamos»

| Variable | % (IC 95%) | Valor de p* | % (IC 95%) | Valor de p* | Dif. % (IC95%) | Valor de p¶ |
|---|---|---|---|---|---|---|
| Satisfacción con la ciudad de Lima como lugar para vivir | 2010 (n=1915) | | 2019 (n=1920) | | 2019-2010 | |
| Población general | 43,3 (41,1-45,6) | | 37,5 (35,4-39,7) | | -5,8 (-8,9 a -2,7) | <0,001 |
| Nivel socioeconómico | | | | | | |
| A/B | 47,4 (43,0-51,9) | | 39,3 (35,7-43,0) | | -8,1 (-13,9 a -2,4) | 0,005 |
| C | 41,8 (38,5-45,1) | 0,109 | 38,3 (34,9-41,8) | 0,155 | -3,5 (-8,3 a 1,3) | 0,152 |
| D/E | 42,2 (38,2-46,3) | | 33,9 (29,8-38,3) | | -8,3 (-14,2 a -2,4) | 0,006 |
| Satisfacción con la calidad del aire de Lima | 2010 (n=1986) | | 2019 (n=1918) | | | |
| Población general | 16,7 (15,1-18,5) | | 10,2 (8,9-11,7) | | -6,5 (-8,7 a -4,3) | <0,001 |
| Nivel socioeconómico | | | | | | |
| A/B | 17,8 (14,6-21,5) | | 11,2 (9,0-13,7) | | -6,6 (-10,8 a -2,5) | 0,002 |
| C | 13,7 (11,6-16,2) | 0,005 | 12,2 (10,0-14,7) | 0,001 | -1,6 (-4,9 a 1,7) | 0,354 |
| D/E | 20,2 (17,1-23,7) | | 5,5 (3,8-8,0) | | -14,7 (-18,6 a -10,8) | <0,001 |
| Satisfacción con los niveles de ruido de Lima | 2010 (n=1891) | | 2019 (n=1914) | | | |
| Población general | 16,8 (15,1-18,5) | | 7,7 (6,6-9,0) | | -9,0 (-11,1 a -7,0) | <0,001 |
| Nivel socioeconómico | | | | | | |
| A/B | 15,0 (12,1-18,5) | | 9,7 (7,8-12,2) | | -5,3 (9,1 a -1,4) | 0,008 |
| C | 16,4 (14,0-19,0) | 0,238 | 7,7 (6,0-9,8) | 0,010 | -8,7 (-11,9 a -5,6) | <0,001 |
| D/E | 18,8 (15,8-22,3) | | 4,9 (3,3-7,3) | | -13,9 (-17,7 a -10,1) | <0,001 |
| Satisfacción con las áreas verdes de Lima | 2010 (n=1910) | | 2019 (n=1918) | | | |
| Población general | 34,1 (32,0-36,2) | | 16,5 (14,9-18,3) | | -17,6 (-20,2 a -14,9) | <0,001 |
| Nivel socioeconómico | | | | | | |
| A/B | 45,2 (40,8-49,6) | | 21,1 (18,3-24,3) | | -24,0 (-29,4 a -18,6) | <0,001 |
| C | 31,9 (28,9-35,1) | <0,001 | 15,7 (13,2-18,5) | <0,001 | -16,2 (-20,3 a -12,2) | <0,001 |
| D/E | 27,9 (24,4-31,7) | | 10,9 (8,4-14,1) | | -17,0 (-21,7 a -12,4) | <0,001 |
| Satisfacción con el espacio público de Lima | 2015 (n=1907) | | 2019 (n=1916) | | | |
| Población general | 31,5 (29,4-33,6) | | 23,8 (21,9-25,8) | | -7,7 (-10,5 a -4,9) | <0,001 |
| Nivel socioeconómico | | | | | | |
| A/B | 35,4 (32,0-38,9) | | 26,9 (23,7-30,3) | | -8,5 (-13,3 a -3,8) | <0,001 |
| C | 32,5 (28,9-36,0) | <0,001 | 20,9 (18,1-24,0) | 0,030 | -11,6 (-16,2 a -7,0) | <0,001 |
| D/E | 24,4 (20,6-28,2) | | 23,8 (20,2-27,9) | | -0,5 (-6,0 a 4,8) | 0,835 |
| Satisfacción con el espacio público del lugar donde vive | 2015 (n=1915) | | 2019 (n=1918) | | | |
| Población general | 29,6 (27,6-31,7) | | 23,5 (21,6-25,4) | | -6,1 (-8,9 a -3,4) | <0,001 |
| Nivel socioeconómico | | | | | | |
| A/B | 41,4 (37,9-45,0) | | 33,2 (29,7-36,7) | | -8,2 (-13,2 a -3,3) | 0,001 |
| C | 24,7 (21,5-28,0) | <0,001 | 16,9 (14,2-19,6) | <0,001 | -7,9 (-12,1 a -3,6) | <0,001 |
| D/E | 18,6 (15,2-22,1) | | 19,2 (15,6-22,8) | | 0,5 (-4,4 a 5,5) | 0,827 |
| Satisfacción con el recojo de basura en Lima | 2010 (n=1910) | | 2019 (n=1917) | | | |
| Población general | 42,3 (40,1-44,5) | | 13,1 (11,7-14,7) | | -29,2 (-31,8 a -26,5) | <0,001 |
| Nivel socioeconómico | | | | | | |
| A/B | 51,6 (47,1-56,0) | | 19,2 (16,4-22,3) | | -32,4 (-37,7 a -27,0) | <0,001 |
| C | 38,9 (35,7-42,2) | <0,001 | 10,5 (8,5-12,9) | <0,001 | -28,4 (-32,3 a -24,5) | <0,001 |
| D/E | 39,7 (35,7-43,7) | | 8,3 (6,1-11,2) | | -31,3 (-36,0 a -26,6) | <0,001 |
| Satisfacción con el acceso y calidad del agua de Lima | 2016 (n=1907) | | 2019 (n=1915) | | | |
| Población general | 30,5 (28,4-32,6) | | 22,0 (20,2-23,9) | | -8,4 (-11,2 a -5,6) | <0,001 |

*Continua en la siguiente página*

**Anexo 2.** Satisfacción y percepciones sobre aspectos de la calidad de vida urbana de los residentes en Lima Metropolitana, según niveles socioeconómicos y años, en la encuesta «Lima Cómo Vamos»

| Variable | % (IC 95%) | Valor de p* | % (IC 95%) | Valor de p* | Dif. % (IC95%) | Valor de p¶ |
|---|---|---|---|---|---|---|
| Nivel socioeconómico | | | | | | |
| A/B | 33,9 (30,5-37,5) | | 27,3 (24,1-30,8) | | -6,6 (-11,4 a -1,8) | 0,007 |
| C | 29,8 (26,5-33,3) | 0,023 | 21,6 (18,8-24,7) | <0,001 | -8,1 (-12,6 a -3,6) | <0,001 |
| D/E | 26,7 (23,0-30,7) | | 14,7 (11,8-18,3) | | -11,9 (-17,0 a -6,9) | <0,001 |
| Satisfacción con los servicios de salud de Lima | 2010 (n=1896) | | 2019 (n=1909) | | | |
| Población general | 25,6 (23,7-27,6) | | 14,7 (13,2-16,4) | | -10,9 (-13,4 a -8,3) | <0,001 |
| Nivel socioeconómico | | | | | | |
| A/B | 21,5 (18,1-25,5) | | 12,5 (10,2-15,1) | | -9,1 (-13,5 a -4,6) | <0,001 |
| C | 25,6 (22,8-28,7) | 0,026 | 14,2 (11,9-16,9) | 0,011 | -11,4 (-15,3 a -7,6) | <0,001 |
| D/E | 28,9 (25,3-32,7) | | 18,8 (15,5-22,6) | | -10,1 (-15,3 a -5,0) | <0,001 |
| Percepción de la inseguridad ciudadana como uno de los tres problemas más importantes de la ciudad | 2010 (n=1920) | | 2019 (n=1920) | | | |
| Población general | 73,5 (71,5-75,5) | | 82,2 (80,5-83,9) | | 8,7 (6,1 a 11,3) | <0,001 |
| Nivel socioeconómico | | | | | | |
| A/B | 81,5 (77,8-84,7) | | 85,1 (82,3-87,6) | | 3,7 (-0,7 a 8,0) | 0,099 |
| C | 72,2 (69,1-75,1) | <0,001 | 82,1 (79,1-84,7) | 0,008 | 9,8 (5,8 a 13,9) | <0,001 |
| D/E | 68,8 (64,9-72,5) | | 78,0 (74,1-81,6) | | 9,2 (3,9 a 14,6) | 0,001 |
| Percepción de que Lima es una ciudad segura | 2010 (n=1916) | | 2019 (n=1920) | | | |
| Población general | 17,7 (16,0-19,5) | | 11,4 (9,9-12,8) | | -6,4 (-8,6 a -4,2) | <0,001 |
| Nivel socioeconómico | | | | | | |
| A/B | 14,8 (11,7-18,0) | | 10,7 (8,4-13,0) | | -4,1 (-8,0 a -0,2) | 0,04 |
| C | 16,7 (14,2-19,2) | 0,007 | 11,4 (9,1-13,7) | 0,684 | -5,3 (-8,7 a -1,9) | 0,002 |
| D/E | 21,8 (18,4-25,2) | | 12,4 (9,4-15,3) | | -9,4 (-13,9 a -4,9) | <0,001 |
| Percepción de seguridad en la zona de residencia | 2010 (n=1916) | | 2019 (n=1920) | | | |
| Población general | 16,0 (14,4 -17,7) | | 21,1 (19,3-23,0) | | 51,2 (2,6 a 7,6) | <0,001 |
| Nivel socioeconómico | | | | | | |
| A/B | 27,1 (23,3-31,2) | | 25,4 (22,3-28,8) | | -1,6 (-6,7 a 3,5) | 0,530 |
| C | 12,9 (10,8-15,3) | <0,001 | 18,7 (16,1-21,7) | 0,002 | 5,9 (2,3 a 9,5) | 0,001 |
| D/E | 11,3 (8,9-14,1) | | 18,3 (15,1-22,1) | | 7,1 (2,7 a 11,4) | 0,002 |
| Percepción del transporte público como uno de los tres problemas más importantes de la ciudad | 2010 (n=1920) | | 2019 (n=1920) | | | |
| Población general | 55,7 (53,4-57,9) | | 46,2 (44,0-48,4) | | -9,5 (-12,6 a -6,3) | <0,001 |
| Nivel socioeconómico | | | | | | |
| A/B | 66,3 (61,9-70,3) | | 53,4 (49,7-57,1) | | -12,8 (-18,4 a -7,2) | <0,001 |
| C | 55,9 (52,5-59,2) | <0,001 | 42,6 (39,1-46,1) | <0,001 | -13,3 (-18,1 a -8,4) | <0,001 |
| D/E | 46,4 (42,4-50,5) | | 41,2 (36,8-45,7) | | -5,3 (-11,3 a -7,3) | 0,086 |
| Percepción de la contaminación vehicular como el problema medioambiental más importante de la ciudad | 2013 (n=1920) | | 2019 (n=1920) | | | |
| Población general | 74,0 (72,0-75,9) | | 72,3 (70,3-74,3) | | -1,6 (-4,4 a 1,1) | 0,259 |
| Nivel socioeconómico | | | | | | |
| A/B | 80,6 (71,3-89,8) | | 72,4 (69,1-75,7) | | -8,1 (-17,9 a 1,7) | 0,104 |
| C | 74,0 (71,3-76,7) | 0,408 | 72,4 (69,2-75,6) | 0,989 | -1,6 (-5,8 a 2,6) | 0,461 |
| D/E | 73,3 (70,3-76,4) | | 72,1 (68,0-76,1) | | -1,3 (-6,3 a 3,8) | 0,624 |

A/B: corresponde a los mayores niveles socioeconómicos.
D/E: corresponde a los menores niveles socioeconómicos.
IC 95%: intervalo de confianza al 95%; Dif. %: diferencia en puntos porcentuales.
* Chi cuadrado para diferencias entre categorías de cada año incluido.
¶ Prueba t entre el valor del año más antiguo y más reciente.
