# Supplementary material for: Satisfaction and perceptions about aspects of the city that affect health, by socioeconomic status, 2010-2019, in Lima
Source: Rev Peru Med Exp Salud Publica. 2022 Mar 31;39(1):83–90. doi: 10.17843/rpmesp.2022.391.9888 (PMC11397593; doi:10.17843/rpmesp.2022.391.9888)
Supplement: Translation

# Translation: Satisfacción y percepciones sobre aspectos de la ciudad que afectan la salud, por nivel socioeconómico, 2010-2019, en Lima Metropolitana

**Authors:** Victoria Cavero, Akram Hernández-Vásquez, J. Jaime Miranda, Patricia Alata, Mariana Alegre, Francisco Diez-Canseco

**Affiliations:** 1 CRONICAS Centro de Excelencia en Enfermedades Crónicas, Universidad Peruana Cayetano Heredia, Lima, Perú.; 2 Lima Cómo Vamos (LCV), Lima, Perú.

**Keywords:** Salud Urbana, Salud Ambiental, Salud Pública, Calidad de Vida, Satisfacción Personal, Disparidades en el Estado de Salud, Política Pública, Planificación Urbana, América Latina, Encuestas y Cuestionarios

## Abstract

Se buscó caracterizar la satisfacción y percepciones de los residentes de Lima Metropolitana sobre distintos aspectos de la ciudad que pueden afectar su calidad de vida y salud, identificando diferencias por nivel socioeconómico (NSE) y cambios en el tiempo. Se realizó un análisis secundario de la Encuesta «Lima Cómo Vamos» entre 2010 y 2019, reportando los resultados mediante porcentajes, con diferencias entre NSE para cada año y entre años. En 2019 la satisfacción y percepciones eran mayoritariamente desfavorables, y han disminuido hasta en 30 puntos porcentuales con el tiempo. Las personas de NSE más bajos tenían valoraciones más desfavorables y con mayores reducciones en el tiempo. Esta insatisfacción y percepciones desfavorables revelan deficiencias en servicios públicos y condiciones urbanas que podrían afectar negativamente la calidad de vida y salud, haciendo necesarias políticas que reduzcan las brechas socioeconómicas y mejoren la salud de los ciudadanos de Lima Metropolitana.

## INTRODUCCIÓN

La población mundial urbana se ha cuadruplicado desde 1950, convirtiendo a América Latina en una de las regiones más urbanizadas ^1^. Este rápido crecimiento, frecuentemente desprovisto de políticas sociales y de planificación, ha generado ciudades con marcadas inequidades en el acceso a servicios públicos, vivienda, transporte, seguridad o recreación, perjudicando a las poblaciones más pobres y vulnerables ^2^ y afectando su calidad de vida ^3^ y salud. Las enfermedades cardiovasculares, inmunológicas, asma, cáncer y obesidad tienen mayor prevalencia en áreas urbanas ^4^, y condiciones como la contaminación o el limitado acceso a servicios de agua y manejo de residuos representan riesgos para la salud ^5^. Estas consecuencias, sin embargo, no son homogéneas en las ciudades latinoamericanas, donde el nivel socioeconómico (NSE) media la susceptibilidad de la población a la contaminación ambiental, y en el que los más pobres tienen mayor riesgo de mortalidad por enfermedades respiratorias ^6^. Asimismo, si bien el acceso a áreas verdes se asocia con mayor actividad física y bienestar mental, y menor contaminación; estos beneficios están mediados por condiciones que, como la inseguridad, afectan el uso efectivo de estas áreas ^7^. 

Lima Metropolitana ha crecido también con escasa planificación y marcadas desigualdades, reflejadas en brechas de acceso a servicios de salud o agua potable ^8^. Dado el potencial impacto de estas condiciones en la calidad de vida y salud de los ciudadanos, es importante ubicarlos en el centro de las decisiones para fortalecer políticas públicas urbanas ^9^. Otras encuestas en Lima han explorado la satisfacción con servicios públicos ^10^ o percepciones sobre corrupción ^11^, pero no sobre el impacto de las condiciones urbanas en la salud de los ciudadanos.

El objetivo de este estudio es caracterizar la satisfacción y las percepciones de los residentes de Lima Metropolitana sobre distintos aspectos y servicios de la ciudad que pueden afectar su calidad de vida y salud, identificando diferencias por NSE y cambios en el tiempo, utilizando información de la encuesta «Lima Cómo Vamos» (LCV). El análisis de esta encuesta, que previamente ha informado políticas para Lima Metropolitana ^12^, brindará evidencia valiosa para mejorar las políticas públicas urbanas que promuevan el bienestar y prevengan resultados negativos en salud.

**MENSAJES CLAVE**

Motivaciones para realizar el estudio: las condiciones de vida y desigualdades socioeconómicas urbanas afectan la salud de las personas; por lo que conocer las percepciones y la satisfacción de los individuos permitirá priorizar políticas que mejoren su calidad de vida y su salud.

Principales hallazgos: al revisar los datos de una encuesta aplicada anualmente a residentes de Lima, se encontró muy baja satisfacción con la ciudad y sus servicios públicos; así como percepciones desfavorables sobre seguridad y transporte público. Estas valoraciones han empeorado con los años, especialmente en los niveles socioeconómicos bajos.

Implicancias: estos resultados permitirán priorizar políticas que promuevan el bienestar y prevengan resultados negativos en salud.

## EL ESTUDIO

Se realizó un análisis secundario de la encuesta LCV del 2010 al 2019. Desde el 2010, se investiga a una muestra representativa, diferente cada año, de 2000 residentes en Lima Metropolitana para recoger su satisfacción y percepciones con distintas dimensiones de la ciudad (ej. contaminación, transporte, saneamiento). Los procedimientos de la encuesta, aplicada por el Instituto de Opinión Pública de la Pontificia Universidad Católica del Perú (IOP-PUCP), se detallan en el material suplementario.

Se utilizó el NSE como variable de exposición para analizar las diferencias en satisfacción y percepciones de los ciudadanos. El NSE fue clasificado en tres niveles (A/B [mayores NSE], C y D/E [menores NSE]), según la metodología de cálculo de NSE del IOP-PUCP.

Se seleccionaron 14 preguntas que abordaban la satisfacción y percepciones con dimensiones relacionadas con la salud y que tenían un número suficiente de personas encuestadas en el desagregado de respuestas. Las preguntas pertenecen a las dimensiones satisfacción con la ciudad, seguridad ciudadana, ambiente, espacios públicos, vivienda y servicios públicos. En la Tabla 1 se presentan las variables seleccionadas, cada una de las cuales corresponde a una pregunta de la encuesta. Aquellas de escala Likert con cinco categorías fueron clasificadas en tres categorías para simplificar la interpretación de resultados. Si bien algunas preguntas de la encuesta se aplican desde 2010, otras se han agregado en años posteriores, por recomendación de un panel de especialistas^ (^ ^13^, por lo que para algunas variables, el año de comparación no es 2010 sino 2013, 2015 o 2016.

**Tabla 1:** Variables incluidas en el estudio, incluyendo código en la base de datos, pregunta y años de comparación de la encuesta.

Código	Variable de interés	Pregunta	Años de comparación ^c^
EG3_X	Satisfacción con la ciudad de Lima como lugar para vivir (sí/no) ^a^	En general, ¿Qué tan satisfecho(a) se siente usted con Lima como una ciudad para vivir?	2010 y 2019
MA1F_X	Satisfacción con la calidad del aire de Lima (sí/no) ^a^	¿Cómo califica en general su nivel de satisfacción con los siguientes aspectos que influyen en la calidad de vida de las personas en Lima?	2010 y 2019
MA1D_X	Satisfacción con los niveles de ruido en Lima (sí/no) ^a^	¿Cómo califica en general su nivel de satisfacción con los siguientes aspectos que influyen en la calidad de vida de las personas en Lima?	2010 y 2019
MA1E_X	Satisfacción con las áreas verdes de Lima (sí/no) ^a^	¿Cómo califica en general su nivel de satisfacción con los siguientes aspectos que influyen en la calidad de vida de las personas en Lima?	2010 y 2019
EP5_X	Satisfacción con el espacio público de Lima (sí/no) ^a^	Entendiendo espacio público como los lugares abiertos a todas las personas en la ciudad, ¿Qué tan satisfecho está usted con el espacio público disponible en Lima?	2015 y 2019
EP6_X	Satisfacción con el espacio público del lugar donde vive (sí/no) ^a^	¿Qué tan satisfecho está usted con el espacio público disponible en el lugar donde vive?	2015 y 2019
MA1C_X	Satisfacción con el recojo de basura en Lima (sí/no) ^a^	¿Cómo califica en general su nivel de satisfacción con los siguientes aspectos que influyen en la calidad de vida de las personas en Lima?	2010 y 2019
MA1I_X	Satisfacción con el acceso y calidad del agua de Lima (sí/no) ^a^	¿Cómo califica en general su nivel de satisfacción con los siguientes aspectos que influyen en la calidad de vida de las personas en Lima?	2016 y 2019
EG6E_X	Satisfacción con los servicios de salud de Lima (sí/no) ^a^	¿Cómo califica su nivel de satisfacción con los servicios de salud que existen en la ciudad de Lima?	2010 y 2019
EG7_1	Percepción de la inseguridad ciudadana como uno de los tres problemas más importantes de la ciudad (sí/no) ^a^	De la siguiente lista ¿cuáles cree que son los tres problemas más importantes que afectan la calidad de vida en la ciudad de Lima?	2010 y 2019
EG7_2			
EG7_3			
EG4_X	Percepción de que Lima es una ciudad segura (sí/no) ^b^	Actualmente, con relación a la violencia y la delincuencia, ¿diría que Lima es segura?	2010 y 2019
VS1_X	Percepción de seguridad en la zona de residencia (sí/no) ^b^	Actualmente, con relación a la violencia y la delincuencia, ¿diría que el lugar donde usted vive es seguro?	2010 y 2019
EG7_1	Percepción del transporte público como uno de los tres problemas más importantes de la ciudad (sí/no) ^a^	De la siguiente lista ¿cuáles cree que son los tres problemas más importantes que afectan la calidad de vida en la ciudad de Lima?	2010 y 2019
EG7_2			
EG7_3			
MA2_1_1	Percepción de la contaminación vehicular como el problema medioambiental más importante de la ciudad (sí/no) ^a^	De los temas relacionados con la gestión ambiental, ¿cuáles cree usted que son los tres problemas ambientales más graves?	2013 y 2019

a Las preguntas son realizadas con una escala de 1 al 5, siendo 1 «Muy insatisfecho» y 5 «Muy satisfecho». Se categorizó como «Sí» cuando la persona manifestó estar satisfecha (4-5) y «No» cuando la persona respondió estar insatisfecha (1-2) o indiferente (3).

b Las preguntas son realizadas con una escala de 1 al 5, siendo 1 «Nada seguro» y 5 «Muy seguro». Se categorizó como «Sí» cuando la persona manifestó sentirse segura (4-5) y «No» cuando la persona respondió sentirse insegura (1-2) o ni segura ni insegura (3).

c Se realizó la comparación entre el año más antiguo y el más reciente según disponibilidad de cada pregunta.

Las características sociodemográficas de los participantes fueron descritas mediante frecuencias. La satisfacción y percepciones se reportaron mediante porcentajes con intervalos de confianza al 95% (IC 95%), para los dos años de comparación. Adicionalmente, se determinaron las diferencias entre NSE para cada año mediante la prueba de chi cuadrado. Se analizaron las diferencias en puntos porcentuales (PP) y sus IC 95% en la satisfacción y percepciones según NSE entre el año más antiguo y el más reciente de cada pregunta, mediante una combinación lineal de las estimaciones comparadas. Según la ficha técnica de la encuesta ^13^ las estimaciones no requieren factores de ponderación ya que la distribución de la muestra fue proporcional a la distribución poblacional según datos del Censo 2007. Un valor p<0,05 fue considerado como estadísticamente significativo. El procesamiento y análisis estadístico se realizó en Stata® v14.2.

Las bases de datos, con información anónima de los participantes, y la ficha técnica de la encuesta son de dominio público y acceso libre en: https://www.limacomovamos.org/data/. Al usar datos secundarios de libre acceso, el presente estudio no fue sometido a revisión por un comité de ética. 

## HALLAZGOS

La Tabla 2 describe las características de los encuestados según los años seleccionados. Durante los 10 años de estudio, las mujeres representaron alrededor de 50% y cada grupo etario, un tercio de los encuestados. La proporción de cada NSE en la muestra ha ido cambiando, habiendo más encuestados en A/B y en C que en D/E durante los últimos años. Las estimaciones junto con sus IC 95% se incluyen en el material suplementario.

**Tabla 2:** Características de las personas encuestadas en «Lima Cómo Vamos».

Característica	2010 n (%)	2013 n (%)	2015 n (%)	2016 n (%)	2019 n (%)
Sexo					
Masculino	956 (49,8)	920 (47,9)	923 (48,1)	925 (48,2)	921 (47,9)
Femenino	964 (50,2)	1000 (52,1)	997 (51,9)	995 (51,8)	999 (52,1)
Grupo etario (años)					
18-29	634 (33,0)	647 (33,7)	650 (33,9)	649 (33,8)	555 (28,9)
30-44	632 (32,9)	619 (32,2)	621 (32,3)	623 (32,5)	611 (31,8)
45 o más	654 (34,1)	654 (34,1)	649 (33,8)	648 (33,7)	754 (39,3)
Nivel socioeconómico					
A/B	486 (25,3)	72 (3,8)	743 (38,7)	707 (36,8)	700 (36,5)
C	857 (44,6)	1023 (53,2)	676 (35,2)	704 (36,7)	747 (39,0)
D/E	577 (30,1)	825 (43,0)	501 (26,1)	509 (26,5)	469 (24,5)
Área interdistrital de residencia					
Lima Centro	728 (37,9)	504 (26,2)	504 (26,2)	504 (26,3)	472 (25,9)
Lima Este	424 (22,1)	512 (26,7)	512 (26,7)	512 (26,7)	528 (29,0)
Lima Norte	384 (20,0)	504 (26,3)	504 (26,3)	504 (26,2)	536 (29,3)
Lima Sur	384 (20,0)	400 (20,8)	400 (20,8)	400 (20,8)	288 (15,8)

A/B: corresponde a los mayores niveles socioeconómicos.

D/E: corresponde a los menores niveles socioeconómicos.

Lima Centro: Lima Cercado, Breña, Rímac, La Victoria, Lince, Jesús María, Pueblo Libre, San Miguel, Magdalena del Mar, San Isidro, Miraflores, Barranco, Santiago de Surco, Surquillo, San Borja y San Luis.

Lima Este: San Juan de Lurigancho, Ate, Chaclacayo, La Molina, Lurigancho-Chosica, El Agustino, Santa Anita y Cieneguilla.

Lima Norte: Ancón, Carabayllo, Comas, Independencia, Los Olivos, Puente Piedra, San Martín de Porres y Santa Rosa.

Lima Sur: Chorrillos, San Juan de Miraflores, Villa María del Triunfo, Villa El Salvador, Lurín, Pachacámac, Punta Hermosa, Punta Negra, San Bartolo, Santa María del Mar y Pucusana.

### Satisfacción con la ciudad, el medioambiente y los servicios públicos brindados

La satisfacción con la ciudad se redujo en 5,8 PP entre 2010 y 2019 (43,3 a 37,5%; p<0,001). Por NSE, la satisfacción en A/B y D/E se redujo aproximadamente 8 PP. En 2010 y 2019, la satisfacción por NSE fue similar (Figura 1).

**Figura 1:** Satisfacción con la ciudad, el medioambiente y los servicios públicos brindados, según nivel socioeconómico y año, Lima, Perú.

*Satisfacción con aspectos relacionados al medioambiente*

La proporción de personas satisfechas con la calidad del aire se redujo en 6,5 PP entre 2010 y 2019 (16,7 a 10,2%; p<0,001). Por NSE hubo cambios en A/B y D/E, con una mayor reducción en D/E. Hubo diferencias en la satisfacción entre NSE en 2010 (p=0,005) y 2019 (p=0,001). En 2010, D/E era el estrato más satisfecho con la calidad del aire, pero en 2019 era el menos satisfecho.

La proporción de personas satisfechas con los niveles de ruido se redujo en 9,1 PP entre 2010 y 2019 (16,8 a 7,7%; p<0,001). Esta satisfacción bajó en todos los NSE, aunque más en D/E. Mientras en 2010 no había diferencias entre NSE (p=0,238), en 2019 la satisfacción fue el doble en A/B que en D/E (9,7 vs. 4,9%; p=0,01) (Figura 1).

*Satisfacción con áreas verdes y espacios públicos*

La proporción de personas satisfechas con las áreas verdes se redujo a la mitad entre 2010 y 2019, pasando de 34,1 a 16,5% (p<0,001) y cayó en todos los NSE. En ambos años hubo diferencias entre NSE, encontrándose que en A/B se sentían más satisfechos, especialmente en 2019, donde A/B duplicaba la satisfacción de D/E (21,1 vs. 10,9%).

La satisfacción con los espacios públicos de Lima y los espacios públicos de la zona de residencia se redujeron en aproximadamente 7 PP entre 2015 y 2019 (31,5 a 23,8%; p<0,001; y 29,6 a 23,5%; p<0,001). Por NSE solo hubo reducciones entre 2015 y 2019 en A/B y C. En ambos años había diferencias entre NSE, siendo A/B el estrato más satisfecho. Asimismo, en A/B más personas estaban satisfechas con los espacios públicos de su zona que con los de Lima, tanto en 2010 (41,2 vs. 35,4%) como en 2019 (33,2 vs. 26,9%). En los NSE C y D/E ocurría lo contrario, más personas estaban satisfechas con los espacios públicos de Lima que con los de su zona en 2010 (C: 24,7 vs. 32,5%; D/E: 18,6 vs. 24,4%) y 2019 (C: 16,9 vs. 20,9%; D/E: 19,2 vs. 23,8%) (Figura 1).

*Satisfacción con servicios públicos*

En cuanto al recojo de basura, la satisfacción se redujo a la tercera parte entre 2010 y 2019, pasando de 42,3 a 13,1% (p<0,001), y siendo similar en todos los NSE. En 2010 y 2019 hubo diferencias entre NSE, siendo A/B el más satisfecho; principalmente en 2019, cuando duplicaba la satisfacción de D/E (19,2 vs. 8,3%).

La satisfacción con el acceso y calidad de agua se redujo en 8,4 PP entre 2016 y 2019 (30,5 a 22,0%; p<0,001) y bajó en todos los NSE, aunque más en D/E comparado con A/B (-11,9 vs. -6,6%). Además, A/B fue siempre el estrato más satisfecho; especialmente en 2019, cuando duplicaba a D/E (27,3 vs. 14,7%).

La satisfacción con los servicios de salud se redujo a casi la mitad entre 2010 y 2019 (25,6 a 14,7%; p<0,001), cayendo en todos los NSE. En el NSE D/E se sentían siempre más satisfechos que en otros NSE (Figura 1).

### Percepción sobre los principales problemas de la ciudad

En 2010 y 2019, los dos problemas percibidos como más importantes para la calidad de vida en Lima fueron inseguridad ciudadana y transporte público.

*Inseguridad ciudadana*

La percepción de la inseguridad ciudadana como uno de los principales problemas de Lima aumentó 8,7 PP entre 2010 y 2019 (73,5 a 82,2%; p<0,001); aunque este aumento fue solo en C y D/E (C: p<0,001; D/E: p=0,001). Por NSE la proporción de personas que lo consideraba un problema principal fue siempre mayor en A/B (81,5 y 85,1%) que otros NSE.

La percepción de Lima como una ciudad segura se redujo en 6,4 PP entre 2010 y 2019 (17,7 a 11,4%; p<0,001), cayendo en todos los NSE, pero más en D/E. En 2010, más personas en D/E compartían esta percepción; pero en 2019 no se encontraron diferencias entre NSE.

La percepción de la zona de residencia como segura aumentó 5,1 PP entre 2010 y 2019 (16,0 a 21,1%; p<0,001). Por NSE, la sensación de seguridad aumentó solo en C y D/E. Además, A/B siempre fue el NSE que se sentía más seguro en su lugar de residencia (Figura 2).

**Figura 2:** Percepción sobre los principales problemas de la ciudad y el medioambiente, según nivel socioeconómico y año, Lima, Perú.

*Transporte público*

La percepción del transporte público como uno de los principales problemas de Lima se redujo en 9,5 PP entre 2010 y 2019 (55,7 a 46,2%; p<0,001), cayendo en los NSE A/B y C. En ambos años más personas en A/B que otros NSE lo consideraban un problema importante. No se observaron diferencias en la percepción de la contaminación vehicular como problema medioambiental más grave (Figura 2).

## DISCUSIÓN

El estudio buscó caracterizar la satisfacción y percepciones, incluyendo cambios en el tiempo, de los residentes de Lima Metropolitana sobre aspectos de la vida urbana que podrían afectar su calidad de vida y salud, con énfasis en las diferencias por NSE. Los resultados principales son tres. Primero, en 2019, la gran mayoría de encuestados estaban insatisfechos y percibían desfavorablemente distintos servicios y características de la ciudad. Segundo, aunque muchas de estas valoraciones ya eran bajas en el año inicial (usualmente 2010), la mayoría empeoraron. Tercero, usualmente las valoraciones no eran homogéneas por NSE, siendo las personas del NSE D/E quienes tenían peores valoraciones y mayores reducciones en su satisfacción a lo largo del tiempo. Estos hallazgos sugieren que las mejoras en los indicadores de desarrollo humano de Lima ^14^ no incrementaron la satisfacción de sus ciudadanos y que algunas brechas entre NSE, en lugar de acortarse, habrían aumentado.

En comparación a ciudades como Santiago de Chile ^15^ o Bogotá ^16^, la satisfacción de los limeños con la ciudad y sus servicios es bastante menor. La satisfacción con Santiago de Chile en 2007 fue mayor a 80%, y ha aumentado con los años ^15^; mientras que en Lima no llega a 40%. En Bogotá, la satisfacción con los servicios de agua y recojo de basura fueron 84 y 65%, respectivamente ^16^; bastante más elevadas que el 25 y 15% de Lima. En otras ciudades de la región el reconocimiento de los derechos a un mejor entorno urbano se ha posicionado muchos años antes que en Lima, lo que puede haber generado mejores condiciones y servicios, conllevando ciudadanos más satisfechos.

De todos los temas explorados, la insatisfacción fue más alta en relación con el ruido y la calidad del aire, con peores valoraciones en el tiempo y entre los más pobres. La primera se condice con que la contaminación sonora en Lima excede los estándares nacionales de calidad ambiental para ruido ^17^. Sin embargo, los datos disponibles sobre calidad del aire indican que esta ha mejorado en 60% en la última década; aunque los valores de material particulado (PM10 y PM2,5) aún exceden los máximos permitidos ^18^. Otro resultado relevante es la baja satisfacción con las áreas verdes, que expresa su gran déficit, ya que Lima está entre el 5% de capitales con menos áreas del mundo ^19^.

La baja satisfacción alcanza también a los servicios de agua y recojo de basura. Primero, Lima sufre estrés hídrico crónico, con 20% de limeños sin acceso diario a agua potable ^20^. La mayor insatisfacción en el NSE D/E confirma la urgencia de políticas que reduzcan esta brecha de acceso al agua. Segundo, Lima es la ciudad con mayor generación de basura del Perú, pero tiene varias municipalidades sin programas para el manejo de residuos ni servicios adecuados de limpieza pública ^21^. Finalmente, la satisfacción con los servicios de salud es también baja, 15% en 2019, y ha disminuido en todos los NSE; siendo casi la cuarta parte que la de Bogotá en 2019 ^16^. La baja calidad de los servicios de salud ha sido reportada por la Defensoría del Pueblo ^22^, asociada con problemas de abastecimiento y atención. Finalmente, aunque la criminalidad en Lima ha disminuido con los años ^23^, la inseguridad continúa siendo un problema prioritario. Asimismo, la mayor seguridad en la zona de residencia podría indicar mayor confianza con los vecinos o mejores gestiones de municipalidades distritales.

Son indispensables políticas para reducir las brechas y mejorar los servicios y problemas explorados por sus potenciales impactos en la salud. Ejemplos de estas acciones serían reducir la contaminación del aire, asociada con mayor mortalidad cardiorrespiratoria en Lima ^24^ y mejorar los servicios de agua potable, para atenuar el riesgo de enfermedades gastrointestinales, dengue o caídas al transportar agua al hogar, especialmente en los NSE más bajos ^25^.

Son fortalezas del estudio analizar una encuesta realizada anualmente, por una década, con los mismos instrumentos y metodología, a muestras representativas de una megaciudad. Una limitación es que no todos los resultados del 2019 se comparan con los del 2010, ya que algunas preguntas se incluyeron en años posteriores. Sin embargo, el año de comparación es casi siempre 2010 y en los otros casos existe una diferencia de años suficiente para esperar cambios en el tiempo. Por otro lado, se realizó una recategorización de las escalas Likert de cinco categorías a tres categorías, para simplificar la interpretación de los resultados. Aunque podría generarse un error de medición no diferencial, los informes anuales de la encuesta LCV utilizan también esta recategorización.

Los resultados visibilizan una insatisfacción elevada y en aumento con distintos aspectos de la ciudad, así como brechas por NSE que se amplían con el tiempo. Estos resultados pueden orientar las prioridades de los decisores de políticas, quienes tienden a fijarse más en mejoras de condiciones materiales, como saneamiento o espacios públicos, pero que, como evidenciamos, no derivan en ciudadanos más satisfechos. Desde una mirada de salud pública, es necesario que las autoridades garanticen políticas que mejoren las condiciones de vida urbana, promoviendo el bienestar de los ciudadanos y previniendo consecuencias negativas en salud.
